# Underserved Latinas' Perceptions and Implications Around Breast Cancer Risk Assessment

**DOI:** 10.1002/cam4.71591

**Published:** 2026-03-05

**Authors:** Jane Q. Yap, Jhenitza P. Raygoza, Valentina Hernandez, Crystal Gonzalez, Celine Vachon, Jessica D. Austin

**Affiliations:** ^1^ Department of Quantitative Health Sciences Mayo Clinic Jacksonville Florida USA; ^2^ Biomedical Ethics Research Program Mayo Clinic Rochester Minnesota USA; ^3^ Department of Quantitative Health Sciences, Division of Epidemiology Mayo Clinic Scottsdale Arizona USA; ^4^ Department of Integrated Nutrition Services and Collaborative Research Mountain Park Health Center Phoenix Arizona USA; ^5^ Department of Quantitative Health Sciences, Division of Epidemiology Mayo Clinic Rochester Minnesota USA

**Keywords:** breast cancer risk assessment, Breast cancer, cancer prevention, focus groups, health behavior, interviews, Latina, perceptions, qualitative research, willingness

## Abstract

**Introduction:**

Breast cancer remains a leading cause of death among Latinas. Although breast cancer risk assessment models exist and show promise in reducing mortality, few studies have elicited women's interest in and perceptions around breast cancer risk assessment, particularly among underserved populations, like Latinas, who are susceptible to disparities in breast cancer outcomes. These disparities stem from factors such as limited access to healthcare services, lower rates of screening utilization, language barriers, and socioeconomic challenges, all of which compound their risk and hinder effective engagement with risk assessment tools. This qualitative study aims to explore Latinas' perspectives regarding breast cancer risk assessment and implications on future behaviors.

**Methods:**

Twenty‐three under‐ or uninsured Latinas aged 45–65 with no personal history of breast cancer took part in either a focus group (*n* = 3, 15 participants) or individual interviews (8 participants). Guided by health behavior theories (Theory of Planned Behavior and Health Belief Model), data were transcribed, translated, and analyzed using a deductive‐inductive approach to thematic content analysis, resulting in four themes.

**Results:**

Latinas expressed high interest in knowing their breast cancer risk and reported that the benefits of knowing one's risk outweighed the harms. Latinas also stated that they would continue screening as recommended if found to be at low risk, but that they would speak with their provider or increase screening frequency if found to be at increased risk. Moreover, Latinas described the pivotal role of providers and social networks in influencing interest and perceptions around breast cancer risk assessment and preventive behaviors.

**Conclusion:**

Underserved Latinas viewed breast cancer risk assessment positively and underscored the need for multilevel, culturally tailored strategies to improve breast cancer risk assessment. This study yielded four distinct themes: (1) Interest and Awareness of Personal Breast Cancer Risk, (2) Benefits and Barriers of Knowing Personal Breast Cancer Risk, (3) Screening Implications of Knowing Personal Breast Cancer Risk, and (4) Provider Influence on Breast Cancer Risk Assessment and Behaviors. Potential strategies include efforts to improve provider engagement and communication and leveraging social networks to increase awareness and encourage preventive behaviors.

**Trial Registration:**

NCI‐2023‐00465

## 1 Introduction

Breast cancer (BC) remains a significant public health challenge, with approximately 300,000 new cases and 42,000 deaths projected among women in the US in 2024 [1]. In Arizona, BC incidence mirrors national trends with an age‐adjusted incidence rate of approximately 121.9 per 100,000 women and about 6000 new cases annually [2]. BC is the leading cause of death among Latinas in both the US and Arizona, with a mortality rate of approximately 20.7 per 100,000 women compared to 19.0 per 100,000 for non‐Hispanic White women [3]. Compared to non‐Hispanic White women, Latinas are more prone to receiving diagnoses at younger ages, with a higher proportion of BCs presenting as advanced‐stage diseases and experiencing lower 5‐year survival rates (e.g., 81% for Latinas vs. 90% for non‐Hispanic White women) [3, 4, 5, 6]. These disparities are ascribed to multifaceted factors encompassing inadequate awareness of cancer and screening guidelines, limited access to and utilization of mammography screening services, fear, and logistical obstacles related to insurance coverage and transportation [7, 8, 9, 10, 11, 12].

Mammography screening has been shown to reduce BC mortality by approximately 25% [13]. However, Latinas are less likely to be screened for BC and more likely to die from BC, with only 65%–70% of Latina women aged 50–74 reporting recent mammography compared to 80% of non‐Hispanic White women [6, 14, 15, 16]. Within the last decade, a shift from age‐based to risk‐based BC screening strategies has emerged. The shift toward preventative and personalized medicine has gained prominence with advancements in genetics, risk modeling, and screening practices. Leading guideline organizations, such as the US Preventive Services Task Force and the American College of Radiology, increasingly recommend moving away from a one‐size‐fits‐all approach [17, 18, 19]. Risk‐based approaches to population‐level screening tailored to an individual's genetic, medical, and socio‐behavioral traits hold promise for mitigating disparities among Latinas by directing the appropriate supplementary screening and risk‐reduction measures to women at high risk for BC [20, 21, 22, 23, 24].

Empirically driven BC risk assessment models have been developed that incorporate easily acquired clinical and non‐clinical risk factor data to characterize a woman's chance of developing BC over specific timeframes (e.g., lifetime, 5‐year, or 10‐year periods). For example, the Tyrer‐Cuzick BC prediction model calculates individual BC risk using factors such as family history, hormonal and reproductive history, health behaviors, genetic predispositions (including polygenic risk scores), and breast density [25, 26]. Similarly, the CanRisk model provides comprehensive risk evaluation by integrating these same elements into a user‐friendly platform [27, 28]. These models consider key risk factors, including breast density, which increases BC risk and reduces mammography sensitivity, as well as age, hormonal exposures, and lifestyle factors. In the U.S., risk assessment is increasingly integrated into clinical practice, with these models being used to guide screening decisions, though adoption remains uneven, particularly among underserved populations like Latinas due to barriers such as limited provider training and access to follow‐up care. Federal and state policies support risk communication; for instance, the FDA's 2024 Mammography Quality Standards Act amendments and state breast density notification laws mandate that women be informed about breast density—a significant risk factor—post‐mammography to encourage discussions about personalized risk and additional screening [29, 30]. These risk assessment models enable and enhance targeted prevention and early detection, aiming to lower mortality rates by identifying women who would benefit most from personalized screening and risk‐reduction strategies. However, implementation of BC risk assessment remains limited and may be dependent on one's own risk perception [31].

Health behavior theories, such as the Theory of Planned Behavior (TPB) and Health Belief Model (HBM), provide a framework for understanding women's attitudes and beliefs toward risk assessment and have been applied to similar concepts, such as mammography screening [32, 33]. We utilized these two theories to guide our data collection and analysis by providing a framework for understanding why individuals do or do not engage in a behavior. TPB proposes that an individual's intention to engage in a behavior, such as undergoing mammography or risk assessment, is based on one's attitudes toward the behavior, subjective norms, and an individual's perceived behavioral control [34]. Studies have demonstrated TPB's utility in predicting mammography uptake, showing that positive attitudes and social encouragement increase screening intentions, particularly among ethnic minorities [35, 36], TPB can be used to guide research on human health behavior by analyzing individuals' intentions and actions to explain human behavior regarding mammography screening further. HBM is an exploratory framework that aims to contextualize why individuals change and/or maintain health behaviors [37]. HBM is comprised of several domains relating to individuals' perception of their health, including perceived susceptibility, severity, benefits, and barriers. HBM has been used to explain BC screening adherence, with research indicating that higher perceived susceptibility and benefits correlate with increased mammography use, while barriers like fear or access issues reduce engagement [38] In a study done among Spanish‐speaking Hispanic women, it was found that HBM constructs, particularly perceived benefits and barriers, predicted mammography screening intentions, emphasizing the importance of culturally relevant health education [39] Perceived susceptibility, or risk, has been shown to strongly influence women's engagement in preventive and screening behaviors [40]. Latinas often perceive lower BC risk than non‐Hispanic White women, possibly reflecting culturally misaligned risk communication [41, 42, 43, 44, 45]. Inaccurate risk perceptions can lead to reluctance or avoidance of standard BC screening, further exacerbating disparities in BC outcomes. Effectively implementing risk‐based screening approaches necessitates a clear understanding of attitudes and beliefs as well as communication of women's individual BC risk [46].

Several studies have explored attitudes and beliefs regarding mammography screening, but few have explored Latinas' attitudes and beliefs regarding BC risk assessment and potential implications on screening behavior [47, 48, 49]. Despite this gap, broader research demonstrates the growing role of risk assessment models in personalizing breast cancer prevention. For example, the US WISDOM trial has piloted risk‐stratified screening to optimize early detection, while PROCAS/BC‐Predict in the UK and MyPeBs in Europe have similarly tested risk‐based approaches, showing promise in tailoring screening to individual risk profiles [29, 50, 51]. These initiatives highlight the potential of risk assessment to refine clinical practice, yet their success hinges on public acceptance. Studies examining women's perceptions in the general population reveal a spectrum of responses—ranging from enthusiasm for personalized insights to concerns about complexity or reliability [30]. Among underserved groups, including ethnic minorities like Latinas, research points to additional barriers such as limited health literacy, mistrust in medical systems, and inequities in access to follow‐up care [48, 52, 53, 54, 55]. These findings underscore the need to explore how cultural and social factors shape attitudes toward risk assessment, particularly in populations underrepresented in prior studies. Understanding how risk assessment is perceived and downstream influences on screening uptake remains a critical, yet understudied, area. The primary objective of this study is to elicit Latinas' attitudes and beliefs toward risk communication and its impact on screening behaviors.

## 2 Materials and Methods

### 2.1 Recruitment

This study was performed in partnership with Mountain Park Health Center (MPHC)—a large Federally Qualified Health Center (FQHC)—located in Maricopa County, Arizona. FQHCs have historically served as safety‐net health centers for under‐ or uninsured community members. MPHC has nine sites in and around Phoenix, Arizona. In 2023, MPHC provided primary care to over 104,000 patients in the community. Over 70% of MPHC patients identify as Latino/a, 70% of patients earn below 200% of the federal poverty rate, and approximately 32% of adult patients are uninsured. MPHC also provides on‐site mammography; in 2023 over 2000 mammograms were completed at the MPHC mammography suite.

A bilingual study coordinator partnered with two Promotoras, or community health workers, employed by MPHC to identify and recruit prospective participants to partake in a focus group or individual interview. The study's eligibility criteria included self‐identified Latinas aged 45–65 years, with no personal history of BC, and under‐ or uninsured at the time of enrollment. The selected age range was intended to align with national guidelines that emphasize the importance of screening in this demographic. This approach aims to optimize screening participation while reducing potential variability in uptake among younger and older populations. The age range was capped at 65 to avoid recruiting Medicare recipients, as the primary study focus was on under‐ and uninsured populations. Potential participants were approached in the mammography clinic and provided information about the study, including a flyer to share with other women who may be interested. Promotoras referred interested participants to a bilingual member of the study team to confirm eligibility, gather consent, and schedule the focus group or interview. Additional recruitment efforts included community‐based events (e.g., health fairs) directed at underserved Latina populations and word of mouth through friends and family. All participants received a $50 cash card remuneration for their time participating in the study. The study was reviewed and approved by the Mayo Clinic Institutional Review Board.

### 2.2 Data Collection

A trained bilingual member of the study team conducted focus groups and semi‐structured interviews in English or Spanish using two separate but complementary guides (see Supporting Information). The HBM and TPB were used to shape the data collection and analysis, offering a robust framework to elucidate the motivations behind individuals' engagement or disengagement in a behavior. Informed by HBM and TPB, the focus group and interview guides broadly explored Latinas' perspectives of knowing their BC risk, including benefits and barriers and implications on future screening. The guides also included a hypothetical vignette that explored different scenarios of a 45‐year‐old woman, Mrs. Hernandez, and elicited what advice participants would give based on each scenario to explore how risk information would influence behavior change. Focus groups occurred in person at an MPHC clinic site and included a trained bilingual moderator and notetaker(s). The moderator and notetakers took post‐focus group meeting notes to capture the high‐level findings. The groups responded well, and the moderator would call individuals to ensure equal representation. The focus groups averaged approximately 60 min in duration and interviews, which were conducted via phone call, averaged 30 min in length.

### 2.3 Analysis

Focus groups and interviews were audio recorded, translated, if applicable, and professionally transcribed and cleaned to retain anonymity. Transcripts were further reviewed by a bilingual member of the study team for accuracy prior to analysis. All qualitative analysis was performed using Delve software [56]. Two members trained in qualitative analysis performed thematic content analysis using a deductive‐inductive approach [57, 58]. Each transcript was reviewed in its entirety without coding. An initial deductive code structure was developed based on the HBM and TPB and applied to 2 randomly selected transcripts (1 focus group and 1 solo interview) during an initial open coding session. As new ideas were constructed, inductive themes were considered, allowing the codebook to evolve. The revised codebook was applied to the remaining transcripts independently before coding discrepancies were resolved through weekly consensus meetings. Findings were discussed with the larger study team. Thematic analysis continued until no new ideas were identified. Data collection continued until redundancy or repetition in the data was observed. Given the exploratory nature of this study, we provide a semantic narrative analysis with the intent to directly describe the perspective and experiences of our participants. Following the guidelines for reporting qualitative research [59], we present representative quotes with minimal alterations for readability in semantic style.

## 3 Results

We conducted three focus groups (*N* = 15) and eight individual interviews. Table 1 provides the study population demographics. The mean age of participants was 56, with 96% Spanish speakers; 74% were recruited by Promotoras prior to their mammography screening appointment, 17% were recruited at community events, and 9% were recruited via word of mouth. Four themes were identified that capture implications and perceptions around BC risk assessment in this population and are presented below. These major themes have been organized by title, definition, and exemplary quote in Table 2.

**TABLE 1:** Study demographics.

Participant	Age (years)	Language	Recruitment modality
Focus Group 1, Interviewee 1	58	Spanish	MPHC CHW
Focus Group 1, Interviewee 2	52	Spanish	MPHC CHW
Focus Group 1, Interviewee 4	51	Spanish	Community recruitment
Focus Group 1, Interviewee 5	50	Spanish	Community recruitment
Focus Group 1, Interviewee 6	59	Spanish	MPHC CHW
Focus Group 2, Interviewee 1	50	Spanish	Word of Mouth
Focus Group 2, Interviewee 2	63	Spanish	MPHC CHW
Focus Group 2, Interviewee 3	50	Spanish	MPHC CHW
Focus Group 2, Interviewee 4	46	Spanish	MPHC CHW
Focus Group 3, Interviewee 1	58	Spanish	MPHC CHW
Focus Group 3, Interviewee 2	63	Spanish	MPHC CHW
Focus Group 3, Interviewee 3	58	Spanish	MPHC CHW
Focus Group 3, Interviewee 4	63	Spanish	Word of Mouth
Focus Group 3, Interviewee 5	45	Spanish	MPHC CHW
Focus Group 3, Interviewee 6	65	Spanish	MPHC CHW
Interviewee C1	56	Spanish	Community recruitment
Interviewee C2	47	English	MPHC CHW
Interviewee C3	65	Spanish	MPHC CHW
Interviewee C4	60	Spanish	MPHC CHW
Interviewee C5	55	Spanish	MPHC CHW
Interviewee C6	61	Spanish	MPHC CHW
Interviewee C7	56	Spanish	Word of Mouth
Interviewee C8	60	Spanish	MPHC CHW

*Note:* Study demographics organized by participant identifier, age in years, language spoken during interview, and recruitment modality.

**TABLE 2:** Four major themes.

Major theme	Definition	Exemplary quote
*Theme 1*: Interest and awareness of personal breast cancer risk	Participants expressed strong interest in learning their lifetime BC risk but lacked awareness and understanding of the term, highlighting the critical need for improved health literacy to empower informed preventive health actions, particularly influenced by personal connections to BC and media exposure	“Now, [I get mammograms] due to need, because there are people in my house that are suffering from it and alert you (…) I'm always reading because sometimes, it's just a lack of information that prevents you from doing it.”
*Theme 2*: Benefits and Barriers of knowing personal breast cancer risk	Participants recognized that knowing their BC risk could drive preventive actions like regular screening, early detection, and informed treatment options, but cited logistical barriers (e.g., transportation, childcare), cultural‐linguistic discordance, and emotional concerns (e.g., sadness, anxiety) as challenges, while still universally supporting the use of risk assessment tools and advocating for greater community education on BC risk and screening.	“If you don't know, you're not informed (…) Many people believe that it's going to happen by the mere fact of being informed of it. For example, if you look for many things about cancer, I'll get cancer (…) but I don't agree. The more informed you are, the more you can prevent (…) If I were Mrs. Hernandez, I would do it.”
*Theme 3*: Screening implications of knowing personal breast cancer risk	Participants expressed that knowing their personal BC risk would prompt preventive behaviors, such as more frequent mammograms and prompt consultation with a doctor, particularly in scenarios of high risk, with or without symptoms, underscoring a proactive approach to screening and medical advice regardless of perceived risk or symptom presence	“[If she were symptomatic but low risk] It's the same; either low or high risk, it's still a risk. She needs to get checked.”
*Theme 4*: Provider Influence on Breast Cancer Risk Assessment and Behaviors	Participants indicated that a high BC risk result from an online assessment tool would prompt them to consult their doctor about more frequent mammograms and follow provider recommendations closely, with many relying entirely on explicit provider guidance for follow‐up actions and noting that effective patient‐provider communication significantly influenced their health behaviors	“I think that, if they [risk assessment tool and providers] told me that it [mammography screening] had to be done every 6 months or more often, I would do it. Whatever they told me to do, I would do.”

*Note:* Study major themes organized by major theme title, definition, and an example quote.

### 3.1 Theme 1: Interest and Awareness of Personal Breast Cancer Risk

Participants were asked if they would like to know their lifetime risk for BC. Almost all participants were interested in knowing their risk, but the majority reported that they did not know their personal BC risk. Additionally, participants were unsure what the term “lifetime risk for BC” meant, which was defined in the interviews as “a percentage given to patients that they can use to know at what percentage they would be at risk for BC.” In fact, few participants stated that their doctor or other healthcare professional told them about their lifetime risk of BC or explained what that meant. A common sentiment throughout the interviews was the importance of increased health literacy and its impact on future preventative health actions. The majority of cohort participants stated that additional knowledge meant the opportunity to take more preventative health actions, which all agreed was crucial.

Now, [I get mammograms] due to need, because there are people in my house that are suffering from it and alert you (…) I'm always reading because sometimes, it's just a lack of information that prevents you from doing it. Focus Group 3, Interviewee 1

Many participants stated that knowing someone with BC, especially family members or friends, would motivate them to determine their BC risk. Participants also recalled hearing about BC on television or through media resources about celebrities having BC. They felt these outlets amplified the importance of figuring out their BC risk.

There was a case of the actress Angelina Jolie; she said she had a high risk of developing cancer. Then, according to the news, she removed everything where cancer could develop, uterus, breasts. She removed everything. That's what I heard in the news, because she had a very high risk of having cancer. Focus Group 2, Interviewee C1

### 3.2 Theme 2: Benefits and Barriers of Knowing Personal Breast Cancer Risk

Participants were asked about the benefits and barriers of knowing their BC risk. Most participants stated that knowing their BC risk could help prevent BC by making them more likely to get regular screening. Additional benefits included understanding personal BC risk, early diagnosis and detection, more effective and wide‐ranging treatment options, informing screening behaviors, and peace of mind.

The advantages of knowing the risk are those: to be forewarned, to be informed, and to do the tests regularly. Become aware of the fact that you have to take tests more often in case cancer shows up.

Focus Group 2, Interviewee 1

When speaking about the potential barriers to knowing their BC risk, participants noted logistical barriers such as transportation and childcare. Many also noted the lack of cultural and linguistic concordance as a barrier.

Sometimes we don't seek help due to the language, because sometimes we're ashamed to ask or we're ashamed because we don't feel anything, there are many barriers for a lot of people. Focus Group 1, Interviewee 4

The majority described knowing one's risk could make them feel sad, worried, and anxious about the future. Importantly, no participant explicitly stated they did not want to know about BC risk. This sentiment was confirmed in response to the vignette scenarios. When participants were asked if Mrs. Hernandez should use a tool to identify her percentage of BC risk, the majority stated that she should. Additionally, participants stated that they personally would complete an online risk assessment tool despite feelings of fear or worry.

If you don't know, you're not informed (…) Many people believe that it's going to happen by the mere fact of being informed of it. For example, if you look for many things about cancer, I'll get cancer (…) but I don't agree. The more informed you are, the more you can prevent (…) If I were Mrs. Hernandez, I would do it. Focus Group 2, Interviewee 4

You know that in those cases, you don't even know what to do, but simply to tell her to keep trying, to be strong. To believe in the medicine and to believe in herself; that she's going to overcome that disease (…) What betrays us is the mind. We feel our lives go away when they tell us that (…) I think that the person has to move ahead, continue with the doctor. Interviewee C1

Many acknowledged that, in general, their communities, specifically family members, were unaware of their BC risk and that more should be done to increase knowledge of BC and risk awareness among these populations. Participants also discussed the importance of educating women about BC risk and the need for regular screening.

### 3.3 Theme 3: Screening Implications of Knowing Personal Breast Cancer Risk

Participants were also asked questions about how knowing one's personal risk would change their behavior. Preventative health behavior, such as seeking risk information or completing screening, was favored regardless of how they perceived their overall BC risk. Most participants stated they would get a mammogram more frequently if they were at increased risk for BC.

I would like it done every six months because, if there's something that can be done, do it. Interviewee C8

This sentiment was echoed in the vignette section. Participants were asked to consider how they would feel and respond if they were in Mrs. Hernandez's situation, given her level of BC risk and the presence of symptoms. In scenarios involving high risk and symptoms, participants indicated they would increase screening frequency and promptly consult a doctor.

If I were Mrs. Hernandez, I would get worried and I would try to do the tests. Not to wait for a year. The mammogram is each year so I would do it every six months, for example. In order to be ready and, in case I got cancer, I would be able to attack it from the beginning (…) So, if you told me that I have a high risk, I would do it every six months or less. I wouldn't mind if they charged me $200. I would go every six months or less. Every four. Focus Group 2, Interviewee 1

In cases of high risk without symptoms, participants similarly emphasized the importance of consulting a doctor, noting that seeking medical advice would be prudent regardless of the absence of symptoms.

[If she were symptomatic but low risk] It's the same; either low or high risk, it's still a risk. She needs to get checked. Focus Group 3, Interviewee 2

### 3.4 Theme 4: Provider Influence on Breast Cancer Risk Assessment and Behaviors

Participants said they would consult their doctor about getting a mammogram more frequently if an online risk assessment tool told them they were at high risk for BC. Many also advised Mrs. Hernandez to speak with her doctor immediately about getting a mammogram. Most participants also stated that they would rely on and follow their provider's recommendations, regardless of the follow‐up action. In fact, many stated that they would not actively pursue any additional follow‐up if not explicitly told to by a provider or if their provider said they didn't have to.

I would make an appointment with a doctor so he could order me a mammogram, or I don't know if it can be done in another way to detect cancer. Because if they're telling me that I am at risk, I have to talk to my doctor first and tell him what's happening and he's going to have to decide what's the appropriate treatment. Interviewee C1

I think that, if they [risk assessment tool and providers] told me that it [mammography screening] had to be done every six months or more often, I would do it. Whatever they told me to do, I would do. Interviewee C6

Finally, women noted that provider recommendations made a difference in their health behavior, especially regarding how their providers communicated to them about their BC risk.

The most important thing is to do what the doctor says, to place herself in the hands of the doctor and do whatever needs to be done. Interviewee C8

## 4 Discussion

Our study revealed several key insights and perspectives from Latinas undergoing mammography screening at a FQHC regarding BC risk assessment. We found that there was a significant interest among these women in understanding their individual BC risk and that the benefits of knowing one's risk outweighed the barriers. These findings are consistent with the literature showing Latinas want to know but misperceive their risk [44, 60, 61, 62]. Unique to this study is that all participants were age‐eligible for and regularly received screening mammograms. Per legislative mandate and guideline recommendations, all study participants should have been notified or been told about their personal risk for BC. However, our study identified many participants that were unaware of or not told their personal BC risk. Additionally, many were unaware that an online tool was publicly available for them to assess their risk. These findings likely reflect challenges in risk communication and implementation.

Risk information, such as breast density notifications, is written at higher literacy levels, which may contribute to the challenges women face in seeking and understanding their BC risk [63]. Similar to prior studies, our findings illustrate the role of providers, family, friends, and cultural figures in increasing awareness of BC risk, as a motivator in understanding one's risk and making decisions about their health, including BC screening decisions [64]. The role of providers in risk communication and healthcare decision making is well described. By guiding informed and shared decision making, providers can empower patients to choose screening options like mammography or genetic testing, significantly boosting adherence and proactive health behaviors [64, 65, 66, 67]. However, several studies have identified gaps in patient‐provider communication, especially when there is a lack of cultural and linguistic alignment. Studies have found that intercultural communication may be hindered by prejudice present in medical communities [68, 69, 70, 71, 72]. Despite the availability of risk assessment models, under‐resourced settings like FQHCs may not have the capacity or resources to implement these models in clinical care. As a result, risk assessment is often limited to family history gathering or breast density notification.

Prior studies have outlined concern that informing women of their BC risk would cause harm and may deter them from getting screened [73, 74, 75]. However, our findings support the growing empirical literature that the benefits of knowing one's risk outweigh the potential barriers, including fear and worry [76]. While knowing one's risk could cause worry, women in our study perceived knowing their risk as important for their overall health and well‐being. Additionally, women indicated heightened urgency to speak with a provider and a desire to increase screening frequencies if they were at increased risk, and they would continue with regular screening per their provider's recommendations if they were at low risk. Existing literature supports this notion that fear of the unknown significantly influences health behaviors, leading individuals to favor more frequent screenings as a precautionary measure [77].

Prior studies have shown that women turn to supportive social networks including family and friends when seeking information or advice about healthcare decisions [78, 79, 80, 81, 82]. This was seen in our study, as participants noted supportive social networks encouraged women to seek information on BC risk and engage proactively with health measures. While social networks have the potential to serve as strong facilitators or for BC risk awareness, participants in our study noted limited awareness around the importance of risk and communication about BC more broadly. Understanding the influence of social relationships is crucial in shaping the cancer screening behaviors of Latina. Research indicates that these social dynamics significantly affect health‐related decision‐making and can enhance awareness and participation in screening programs. Women in this cohort also noted that hearing stories of BC screening experiences from those they knew personally influenced their own perspectives on the topic. Efforts to increase awareness around BC risk, such as narrative storytelling may be a beneficial strategy as noted by several studies showing storytelling as well as hearing experiences from personal connections can influence BC risk prevention attitudes and behavior [83, 84, 85, 86, 87].

Our findings suggest that multilevel strategies are needed to engage Latinas in BC risk assessment. Efforts to increase awareness include communication campaigns, digital storytelling, culturally tailored community education campaigns, and engaging community health workers. Increasing education and awareness alone is unlikely to be sufficient. Strategies to promote risk communication and shared decision making among providers as well as engage social networks are necessitated.

### 4.1 Strength and Limitations

A significant strength of this study is the inclusion of underserved, predominately Spanish‐speaking Latinas. Further, we identified these women had not been exposed to BC risk assessment or risk information, despite guideline recommendations and legislative mandates. The homogeneous nature of the sample provided an in‐depth understanding of our population's specific needs and potential targets for future intervention and hypothesis generation. However, it is also important to note our limited ability to generalize our findings to other populations and settings. Most participants were recruited at the time of their screening mammogram or reported a history of receiving a mammogram in the last 2 years.

While this criterion was useful in helping us understand knowledge and awareness of risk assessment, our findings could be biased toward participants who hold positive attitudes and strong motivations toward BC prevention. It is also possible that participants provided perspectives in the context of preventive screening more broadly, given the limited knowledge and awareness around BC risk assessment observed in our sample. While we expected participants to have some exposure to BC risk assessment and/or risk communications per guideline recommendations and legislative mandates, we did not confirm receipt via electronic health record or explore FQHC practices around BC risk assessment. Future studies should explore perspectives among diverse women identified at increased risk, as well as with clinicians and systems to understand multilevel targets to improve the implementation of BC risk assessment practices.

## 5 Conclusion

Underserved Latinas held a strong desire to understand their BC risk, recognizing that the benefits of this information outweigh potential barriers. This interest is consistent with current literature that highlights the importance of providing culturally sensitive and accessible risk assessment information to Latinas from underserved communities to reduce health disparities [88, 89, 90, 91, 92]. Our findings suggest that risk assessment alone is unlikely to impact screening behaviors, and future efforts should explore strategies to improve provider recommendation and communication, as well as engage family and friends. Supportive networks play a significant role in motivating women to seek information and adopt proactive health measures. Moreover, identifying effective ways to communicate this risk assessment information in a way that aligns with cultural needs is imperative to meeting the needs of women in these underserved communities. This is essential to promoting risk‐reducing behaviors and preventative health actions.

## Author Contributions

**Jane Q. Yap:** conceptualization (supporting), data curation (equal), formal analysis (equal), methodology (supporting), software (equal), writing – original draft (lead), writing – review and editing (lead). **Jhenitza P. Raygoza:** data curation (equal), formal analysis (supporting), methodology (supporting), software (equal), writing – review and editing (equal). **Valentina Hernandez:** data curation (supporting), resources (supporting), writing – review and editing (equal). **Crystal Gonzalez:** data curation (supporting), resources (supporting), writing – review and editing (equal). **Celine Vachon:** conceptualization (supporting), writing – review and editing (equal). **Jessica D. Austin:** conceptualization (lead), funding acquisition (lead), investigation (lead), methodology (lead), project administration (lead), resources (lead), supervision (lead), writing – review and editing (equal).

## Funding

This work was supported by the Mayo Clinic Center for Health Equity and Community Engagement Research.

## Ethics Statement

All procedures were in accordance with the ethical standards of the institution and/or national research committee and with the 1964 Helsinki declaration and its later amendments or comparable ethical standards.

## Consent

Informed consent was obtained from all individual participants included in the study.

## Conflicts of Interest

The authors declare no conflicts of interest.

## Supporting information


**Data S1:** Focus Group and Interview Guides in English and Spanish.

**Data S2:** Codebook of themes, subthemes, definitions, exemplar quotes, and codes.

## Data Availability

The data underlying this article cannot be publicly shared due to existing consent and Institutional Review Board constraints. Summary level data and datasets used and/or analyzed without individual data are available from the corresponding author and with permission from community partners who supported this project upon reasonable request.
