# Supplementary material for: Underserved Latinas' Perceptions and Implications Around Breast Cancer Risk Assessment
Source: Cancer Med. 2026 Mar 5;15(3):e71591. doi: 10.1002/cam4.71591 (PMC12963470; doi:10.1002/cam4.71591)
Supplement: Supplementary file 1 — Data S1: Focus Group and Interview Guides in English and Spanish. [file CAM4-15-e71591-s001.pdf]

FOCUS GROUP GUIDE (ENGLISH)

My name is <Your Name> and I work at Mayo Clinic.

Thank you for agreeing to take part in this group discussion. I will ask you to share your thoughts about breast cancer/ mammography screening and a tool used to help women understand their risk of getting breast cancer. There are no right or wrong answers, and we ask that you provide honest feedback. The feedback we receive today will help researchers understand if the tool would be helpful to get women like yourselves to a mammogram appointment. The information that you give me today will not be shared outside of the research team. Participation is voluntary and you can also stop participating at any time.

Do you have any questions for me? We will start recording the session now.

Icebreaker
Let's go around the table and introduce ourselves. Tell us your name, where you are from, and what you enjoy or what makes you proud to be a Hispanic woman?

Questions
1. What are your thoughts about getting a mammogram?
2. Why did you decide to get a mammogram or not to get a mammogram?
3. What makes it hard for you to get a mammogram or medical appointments?

Researchers and providers normally estimate a woman's chance of breast cancer during a medical appointment, but it can be hard for women to get to an appointment.
1. What would be good about knowing your breast cancer risk?
2. What would be bad about knowing your breast cancer risk?
3. How would knowing your breast cancer risk change how you get a mammogram?

Scenario 1: Your name is Mrs. Hernandez. You are 45 years old and have never received a mammogram. A mammogram is an x-ray of each breast to look for cancer. Someone you trust, like a family member or friend, shows you a free online tool that can tell you your estimated lifetime risk of breast cancer using information from women similar to you.

{pass out paper copy of tool}

1. Do you think that women like Mrs. Hernandez would be interested in knowing their risk for breast cancer?
2. Do you think Mrs. Hernandez would complete the tool? Why or why not?
3. What would make Mrs. Hernandez more likely to complete the tool?
    a. Probe: Is it who tells her about the tool? How she completes the tool (online, phone app, in-person with a doctor or community health worker/promotora)?

Scenario 2: Mrs. Hernandez decides she wants to know her risk and fill out the tool. The tool tells her that she may be at high risk for breast cancer, but Mrs. Hernandez is not having any breast symptoms like lumps, nipple discharge or pain.

1. If you were Mrs. Hernandez, how would you feel and what would you do next?
2. If Mrs. Hernandez were your close friend, what advice would you give her? Why?
   a. Do you think Mrs. Hernandez should make an appointment to get a mammogram?
   b. How would your advice change if Mrs. Hernandez was having symptoms?

Scenario 3: Mrs. Hernandez fills out the tool and shows that she may be at low risk for breast cancer, and she is not having any breast symptoms like lumps, nipple discharge or pain.

1. If you were Mrs. Hernandez, how would you feel and what would you do next?
   If Mrs. Hernandez were your close friend, what advice would you give her? Why?
   Probe: Do you think Mrs. Hernandez should make an appointment to get a mammogram?
   How would your advice change if Mrs. Hernandez was having symptoms?

Thank you for your feedback. Researchers and providers normally estimate a woman's chance of breast cancer during a medical appointment, but it can be hard for women to get to an appointment.

4. What would be good about knowing your breast cancer risk?
5. What would be bad about knowing your breast cancer risk?
6. How would knowing your breast cancer risk change how you get a mammogram?

Now, imagine that you have been tasked with getting this form out to Hispanic women in your community.

1. How would you get women in your community to complete this form outside a healthcare setting, like a clinic or hospital?
   a. *Where would they complete it? Would they complete it in-person, by phone through an app, or online?*
2. What would keep women from completing this form and learning their risk in the community?

Those are all the questions we have; do you have any questions? Is there anything else about breast cancer risk or screening mammography you would like to discuss?

FOCUS GROUP GUIDE (SPANISH)

Buenos días, tardes, etc., me llamo <Your Name> y trabajo en Mayo Clinic.

Quisiera darles las gracias por haber aceptado participar en esta conversación grupal. Les pediré que compartan su opinión sobre el cáncer de mama/la mamografía y una herramienta que se utiliza para ayudar a las mujeres a entender su riesgo de contraer dicho cáncer. No hay respuestas correctas o incorrectas y les pedimos que nos den su sincera opinión. La opinión que recibamos hoy ayudará a los investigadores de salud a comprender si la herramienta sería útil para motivar a mujeres como ustedes a hacerse una mamografía. La información que compartan no saldrá del equipo de investigación. La participación es voluntaria y pueden interrumpir su participación en cualquier momento.

¿Tienen alguna pregunta? Comenzaremos a grabar la sesión ahora.

Icebreaker

Preséntense una por una desde sus asientos. Díganos su nombre, de dónde son y qué disfrutan o qué las hace sentirse orgullosas de ser mujeres hispanas.

Preguntas

1. ¿Qué opinan de hacerse una mamografía?
2. ¿Por qué decidieron hacerse una mamografía o no hacerse una?
3. ¿Qué dificulta que se realicen una mamografía o hagan citas médicas?

Situación hipotética 1: Digamos que su nombre es la Sra. Hernandez. Tiene 45 años y nunca se ha realizado una mamografía. Una mamografía son rayos X de ambos senos para detectar cáncer. Digamos que una persona en la que confía, como un familiar o amigo, le muestra una herramienta en línea gratuita que le puede indicar su riesgo estimado de por vida de desarrollar cáncer de mama usando información de mujeres similares a usted.

{pass out paper copy of tool}

1. ¿Creen que mujeres como la Sra. Hernandez estarían interesadas en conocer el riesgo de desarrollar cáncer de mama?
2. ¿Creen que la Sra. Hernandez usaría la herramienta? ¿Por qué sí o por qué no?
3. ¿Qué haría que fuera más probable que la Sra. Hernandez usara la herramienta?
    a. Probe: ¿Creen que dependa de quién le cuente sobre la herramienta? ¿La forma cómo usa la herramienta (en línea, aplicación móvil, en persona con un médico o promotora de salud)?

Situación hipotética 2: La Sra. Hernandez decide que desea conocer el riesgo que corre y llena la información en la herramienta. La herramienta le indica que tal vez tenga riesgo elevado de desarrollar cáncer de mama, pero la Sra. Hernandez no tiene síntomas, como, por ejemplo, protuberancias, secreción del pezón o dolor.

1. Si fueran la Sra. Hernandez, ¿cómo se sentirían y qué harían a continuación?
2. Si la Sra. Hernandez fuera una amiga cercana, ¿qué consejo le darían? ¿Por qué?
   a. ¿Creen que la Sra. Hernandez debería sacar una cita para que le realicen una mamografía?
   b. ¿Cómo cambiaría su consejo si la Sra. Hernandez tuviera algún síntoma?

Situación hipotética 3: La Sra. Hernandez llena la información en la herramienta y esta le indica que tal vez tenga riesgo bajo de desarrollar cáncer de mama y no tiene síntomas tales como protuberancias, secreción del pezón o dolor.

1. Si fueran la Sra. Hernandez, ¿cómo se sentirían y qué harían a continuación?
   Si la Sra. Hernandez fuera una amiga cercana, ¿qué consejo le darían? ¿Por qué?
   Probe: ¿Creen que la Sra. Hernandez debería sacar una cita para hacerse una mamografía? ¿Cómo cambiarían su consejo si la Sra. Hernandez tuviera algún síntoma?

Gracias por sus comentarios. Los investigadores y profesionales de la salud por lo general calculan la probabilidad de desarrollar cáncer de mama en una cita médica, pero puede ser difícil que las mujeres acudan a una cita.

1. ¿Cuáles serían las ventajas de conocer su riesgo de desarrollar cáncer de mama?
2. ¿Cuáles serían las desventajas de conocer su riesgo de desarrollar cáncer de mama?
3. ¿De qué manera cambiaría la forma en que se hacen una mamografía si conocieran el riesgo que tienen de desarrollar cáncer de mama?

Ahora, supongan que se les ha pedido que compartan esta encuesta a las mujeres hispanas en su comunidad.

1. ¿Cómo harían para que las mujeres en su comunidad contestaran esta encuesta aparte de un entorno de atención médica como una clínica u hospital?
   a. *¿Dónde la contestarían? ¿Lo harían en persona, por teléfono, a través de una aplicación móvil o en línea?*
2. ¿Qué impediría que las mujeres contestaran esta encuesta y se enteren del riesgo aparte de un entorno de atención médica como una clínica u hospital?

Esas son todas las preguntas que tenemos. ¿Ustedes tienen alguna pregunta? ¿Hay algo más sobre el riesgo de desarrollar cáncer de mama o sobre la mamografía que quisieran compartir?

INTERVIEW GUIDE (ENGLISH)

My name is <Your Name> and I work at Mayo Clinic.

Thank you for agreeing to take part in this interview. I will ask you to share your thoughts about breast cancer/ mammography screening and a tool used to help women understand their risk of getting breast cancer. There are no right or wrong answers, and we ask that you provide honest feedback. The feedback we receive today will help researchers understand if the tool would be helpful to get women like yourselves to a mammogram appointment. The information that you give me today will not be shared outside of the research team. Participation is voluntary and you can also stop participating at any time.

Do you have any questions for me? We will start recording the session now.

Icebreaker
Tell me about yourself- where you are from, and what you enjoy, or what makes you proud to be a Hispanic woman?

Questions
1. What are your thoughts about getting a mammogram?
2. Why did you decide to get a mammogram or not to get a mammogram?
3. What makes it hard for you to get a mammogram or go to the doctor?

There are tools that can help doctors and researchers to estimate a woman's chance of breast cancer using information provided during a doctor's appointment, but it can be hard for women to get to an appointment.

1. What would be good about knowing your breast cancer risk?
2. What would be bad about knowing your breast cancer risk?
3. Would you be interested in knowing your lifetime risk for breast cancer? Why or why not?
    a. If not, is there anything that would make you more interested in knowing your risk for breast cancer? Why or why not?
4. How would knowing your breast cancer risk change how you get a mammogram?
4. Let's say that there was a tool that women could use to know their estimated risk for breast cancer without going to the doctor. Would you be interested in using such a tool? Why or why not?
    a. How would you prefer to complete the tool? In-person or online or Promotora.

Scenario 1: I'm going to give you a scenario and would like to know what you would do. Mrs. Hernandez is 45 years old and has never had a mammogram. Someone she trusts, like a family member or friend, shows her a free online tool that gives Mrs. Hernandez her estimated lifetime risk of breast cancer using information from women similar to her. Mrs. Hernandez decides she

wants to know her risk and fills out the tool. The tool tells her that she may be at high risk for breast cancer, but Mrs. Hernandez is not having any breast symptoms like lumps, nipple discharge or pain.

1. If you were Mrs. Hernandez, how would you feel? What would you do next?
2. If Mrs. Hernandez were your close friend, what advice would you give her? Why?
    a. Do you think Mrs. Hernandez should make an appointment to get a mammogram?
    b. How would your advice change if Mrs. Hernandez was having symptoms?

Scenario 2: Mrs. Hernandez fills out the tool and is shows that she may be at low risk for breast cancer, and she is not having any breast symptoms like lumps, nipple discharge or pain.

1. If you were Mrs. Hernandez, how would you feel? What would you do next?
2. If Mrs. Hernandez were your close friend, what advice would you give her? Why?
    a. Do you think Mrs. Hernandez should make an appointment to get a mammogram?
    b. How would your advice change if Mrs. Hernandez was having symptoms?

Zoom or in-person: *I'm going to have you look at a form that has questions that researchers and doctors use to estimate the average risk of breast cancer for a woman who has similar risk factors to you.*

Phone: *I'm going to describe to you a form that has questions that researchers and doctors use to estimate the average risk of breast cancer for a woman who has similar risk factors to you. The form asks questions like your age, family history, history of breast biopsy, age at first period, and pregnancy history.*

1. How would you feel completing a form or answering these questions without the doctor?
    a. *Do any of these questions make you uncomfortable?*
2. Are there any questions that are difficult to answer?
3. Would you recommend this form to your family and friends to learn about their estimated risk for breast cancer?
4. Do you think women in your community would find this form to be useful? Why/Why not.

Thank you for your feedback. Now, imagine that you have been tasked with getting this form out to Hispanic women in your community.

1. How would you get women in your community to complete this form outside of a doctor's appointment?
    a. *Where would you go and why?*
2. What would be any barriers for women to complete this form outside of a doctor's appointment?

Those are all the questions we have; do you have any questions? Is there anything else about breast cancer risk or screening mammography you would like to discuss?

INTERVIEW GUIDE (SPANISH)

Buenos días, tardes, etc., me llamo <Your Name> y trabajo en Mayo Clinic.

Quisiera darle las gracias por haber aceptado participar en esta entrevista. Le voy a pedir que que comparte su opinión sobre el cáncer de mama/la mamografía y una herramienta que se utiliza para ayudar a las mujeres a entender su riesgo de contraer dicho cáncer. No hay respuestas correctas o incorrectas y le pido que me de su sincera opinión. La opinión que recibo hoy ayudará a los investigadores de salud a comprender si la herramienta sería útil para motivar a mujeres como usted a hacerse una mamografía. La información que compartan no saldrá del equipo de investigación. Su participación es voluntaria y puede interrumpir su participación en cualquier momento.

¿Tiene alguna pregunta? Comenzaremos a grabar la sesión ahora.

Icebreaker
Hábleme de usted- su nombre, de dónde es, y ¿qué disfruta o qué le hace sentirse orgullosa de ser mujer hispana?

Preguntas
1. ¿Qué opinan de hacerse una mamografía?
2. ¿Por qué decidieron hacerse una mamografía o no hacerse una?
3. ¿Qué dificulta que se realicen una mamografía o hagan citas médicas?

Hay herramientas que pueden ayudar a los doctores e investigadores a dar un promedio o probabilidad del riesgo una mujer tiene de padecer cáncer de seno usando información en una cita de Doctor, pero en veces puede ser difícil para algunas señoras a conseguir una cita.

1. Que seria bueno de saber su riesgo de padecer cáncer de seno?
2. Que seria malo de saber su riesgo de padecer cáncer de seno?
3. Le interesaría saber su riesgo estimado de por vida de padecer cáncer de seno? Por que?
    a. Si no, hay algo que le interesaría mas de saber su riesgo estimado de padecer cáncer de seno? Por que?
4. Como cambiaria su manera de hacerse mamografías si conocer su riesgo estimado de padecer cáncer de seno?
5. Digamos que hay una herramienta que les ayuda a las señoras saber su riesgo estimado de padecer cáncer de seno sin ir al doctor. Le interesaría usar esta herramienta? Porque?
    a. Como prefería completar la herramienta? En persona o con una promotora de salud?

Situación hipotética 1: Le voy a dar un escenario y usted me tiene que decir que haría. Digamos que su nombre es la Sra. Hernandez. Tiene 45 años y nunca se ha realizado una mamografía. Una mamografía son rayos X de ambos senos para detectar cáncer. Digamos que una persona

en la que confía, como un familiar o amigo, le muestra una herramienta en línea gratuita que le puede indicar su riesgo estimado de por vida de desarrollar cáncer de mama usando información de mujeres similares a usted. La Sra. Hernandez decide que desea conocer el riesgo que corre y llena la información en la herramienta. La herramienta le indica que tal vez tenga riesgo elevado de desarrollar cáncer de mama, pero la Sra. Hernandez no tiene síntomas, como, por ejemplo, protuberancias, secreción del pezón o dolor.

1. Si fueran la Sra. Hernandez, ¿cómo se sentirían y qué harían a continuación?
2. Si la Sra. Hernandez fuera una amiga cercana, ¿qué consejo le darían? ¿Por qué?
    a. ¿Creen que la Sra. Hernandez debería sacar una cita para que le realicen una mamografía?
    b. ¿Cómo cambiaría su consejo si la Sra. Hernandez tuviera algún síntoma?

Situación hipotética 2: La Sra. Hernandez llena la información en la herramienta y esta le indica que tal vez tenga riesgo bajo de desarrollar cáncer de mama y no tiene síntomas tales como protuberancias, secreción del pezón o dolor.

1. Si fueran la Sra. Hernandez, ¿cómo se sentirían y qué harían a continuación?
2. Si la Sra. Hernandez fuera una amiga cercana, ¿qué consejo le darían? ¿Por qué?
    a. Probe: ¿Creen que la Sra. Hernandez debería sacar una cita para hacerse una mamografía? ¿Cómo cambiarían su consejo si la Sra. Hernandez tuviera algún síntoma?

Zoom o en-persona: *Voy a pedirle que mire un formulario que tiene preguntas que los investigadores y los médicos usan para estimar el riesgo promedio de cáncer de mama para una mujer que tiene factores de riesgo similares a los suyos.*

Teléfono: *Voy a describirle un formulario que tiene preguntas que los investigadores y los médicos usan para estimar el riesgo promedio de cáncer de mama para una mujer que tiene factores de riesgo similares a los suyos. El formulario hace preguntas como su edad, antecedentes familiares, antecedentes de biopsia de mama, edad en el primer período y antecedentes de embarazo.*

1. ¿Cómo se sentiría completando un formulario o respondiendo estas preguntas sin el médico?
    a. ¿Alguna de estas preguntas te hace sentir incómoda?
2. ¿Hay alguna pregunta que sea difícil de responder?
3. ¿Recomendaría este formulario a su familia y amigos para conocer su riesgo estimado de cáncer de mama?
4. ¿Crees que las mujeres de tu comunidad encontrarían útil este formulario? Por qué/por qué no.

Gracias por sus comentarios. Ahora, imagine que se le ha encomendado la tarea de hacer llegar este formulario a las mujeres hispanas de su comunidad.

1. ¿Cómo conseguiría que las mujeres de su comunidad completaran este formulario fuera de una cita con el médico?
    a. ¿A dónde irías y por qué?
2. ¿Cuáles serían las barreras para que las mujeres completen este formulario fuera de una cita con el médico?

Esas son todas las preguntas que tenemos; ¿Tienes alguna pregunta? ¿Hay algo más sobre el riesgo de cáncer de mama o la mamografía de detección que le gustaría discutir?
