# Supplementary material for: Underserved Latinas' Perceptions and Implications Around Breast Cancer Risk Assessment
Source: Cancer Med. 2026 Mar 5;15(3):e71591. doi: 10.1002/cam4.71591 (PMC12963470; doi:10.1002/cam4.71591)
Supplement: Supplementary file 2 — Data S2: Codebook of themes, subthemes, definitions, exemplar quotes, and codes. [file CAM4-15-e71591-s002.docx]

| THEME | SUBTHEME 1 | SUBTHEME 2 | DEFINITION | EXAMPLE | CODE |
| --- | --- | --- | --- | --- | --- |
| Reasons for getting a mammogram |  |  | Responses to their thoughts about getting a mammogram or the importance/motivation. This is not related to their decision but there could be an overlap. | Early detection, saves lives, felt pain, family history, personal experience, be there for the family. | Reasons (mammogram) |
|  | Family history |  | Observational. Any mention of a family member being diagnosed with any type of cancer. Hearing more about cancer. It is not limited to first degree only. | Instances of family having cancer but there was no involvement with care. Seeing more awareness from people/celebrities diagnosed with cancer. | (Observational) Family history |
|  | Doctor recommendation |  | Statements of following a medical provider’s recommendation to get screened. | “I do my mammogram every time the doctor tells me.” | Doctor recommendation (reasons) |
|  | To be a good example |  | Mentions of supporting, educating, or motivating other women to get a mammogram including family. Social support. | Sharing knowledge with family members or friends. | Good example |
|  | Important for one’s overall health |  | Any mention that getting a mammogram is important for one’s health. | “It is important that we take care of ourselves, our overall well-being.” | Important for health |
|  | Having knowledge or experience with cancer |  | Responses to help a family member, friend, or themselves. Active caregiver or personal role. | Caregiver role. Active role in their care. Family members had cancer and personal experience cancer. | (Active) Caregiver or personal experience |
|  | Benefits outweigh barriers |  | Statements that start by saying a barrier to getting a mammogram but end with a positive perspective. | “At first, I was nervous, or it was painful, but it was worth it. It is important. “ | Benefits outweigh barriers |
|  | Early detection |  | Statements that mention getting a mammogram to detect cancer early. | “It is important for us to screen because the sooner we find something the more likely we can prevent aggressive cancer.” | Early detection (reasons) |
|  | Emotional |  | Statements regarding emotions tied to getting a mammogram, breast cancer risk, knowledge about breast cancer. | “It’s scary and that’s why I don’t want to or it’s scary that’s why I want to. “ | Emotional |
|  | Screening perception |  | Statements about how they, and people around them, perceive breast cancer screening. Statements can include misperceptions about guidelines, procedures, or reasons for getting it. | “You do not need a mammogram if you do not have symptoms.” | Screening perception |
|  | Breast cancer perception |  | Statements about how they and people around them perceive breast cancer. Statements can include misperceptions about cancer, risk, etc. | “Breast cancer = death” | Breast cancer  Perception |
| Decision to get a mammogram |  |  | Specific responses to the decision for why women got a mammogram. | Increased awareness about cancer, being told to get a mammogram, is important for health. | Decisions (mammogram) |
|  | Doctor recommendation |  | The decision to get a mammogram was because the doctor recommended it. | “I was unsure to get a mammogram, but I decided to after the doctor recommended it.” | Doctor recommendation (decision) |
|  | Early detection |  | The decision was because it was important for early detection and survival. | “I decided to get screened because I want to catch it early if it is positive.” | Early detection (decision) |
|  | Family or friend recommendation |  | The decision to get a mammogram was because a family or friend recommended it. | “I decided to get screened because my friend’s mother had tested positive, and she recommended everyone to get screened.” | Family/friend recommendation (decision) |
| Barriers to getting a mammogram |  |  | Anything that makes it challenging for women to get their mammogram | Can include factors of cost, no insurance, lack of resources, not knowing where to go for resources, waiting till something is wrong. | Barriers (mammogram) |
|  | Financial barriers |  | Responses about mammograms being expensive or not covered by insurance. | “I did not have insurance to cover my mammogram.” | Financial barriers |
|  | Cultural myths |  | Any mention of cultural beliefs, myths, or stories. | “The machine smashes your breast like a *tortilla*.” | Cultural myths |
|  | Fear |  | Mention of fear as a barrier to mammogram. | “I am afraid of the pain of the mammogram, so I would rather not do it.” | Fear (barriers) |
|  | Lack of knowledge |  | Mentions of not having the knowledge of where to go to get resources. | “I do not know where to get free mammograms.” | Lack of knowledge (barriers) |
|  | Logistical barrier |  | Logistical barriers and obstacles such as lack of transportation, and other physical barriers or it not being done correctly. | “I cannot find transportation to get to my mammogram appointment.” | Logistical barrier |
| Facilitators to getting a mammogram |  |  | Anything that makes it easier for women to get a mammogram. | Statements can include factors of screening covered by insurance, free, program was available, resources, and having time. | Facilitators (mammogram) |
| Screening |  |  | Anything mentioned regarding screening. | Statements regarding screening. | Screening |
|  | Screening frequency/adherence |  | Any mention of how often they get a mammogram or are told to get a mammogram. | “I get a mammogram done every two years.” | Frequency/adherence |
|  | Screening preferences |  | Mentions of where they would prefer to receive a screening mammogram and why. | “I would prefer getting my mammograms in clinic vs. the mobile units.” | Preference |
|  | Screening modality |  | Any mention of screening modalities outside of screening mammograms. | “I had to do a biopsy to rule out cancer.” | Screening modalities (except mammogram) |
| Breast cancer risk |  |  | Anything mentioned regarding breast cancer risk | Perception of breast cancer risk, factors, perceived barriers, etc. | Breast cancer risk |
|  | Knowledge of breast cancer risk |  | Any mention of being told or being aware of breast cancer risk or probability of getting breast cancer. | “I was told I had high risk of breast cancer.” | Knowledge (BC risk) |
|  | Factors that increase the risk of breast cancer |  | Mention of risk factors that increase one’s chances of getting BC. | “I think I would have high risk of breast cancer because my mom was diagnosed at my current age.” | Factors |
|  | Benefits of knowing individual risk |  | Positive response to knowing one’s individual risk for breast cancer. | “It would be good to know my risk of breast cancer to start screening on time.” | Benefits of knowing |
|  | Barriers to knowing individual risk |  | All-encompassing. Code includes practical barriers to finding out breast cancer risk as well as negative responses to knowing one’s individual risk for breast cancer. | “I would rather not know my risk since I do not have insurance for follow up.” | Barriers (risk) |
|  | Interest in knowing risk |  | Positive or negative responses to women wanting to know their risk for breast cancer. | “I would rather know my risk than not.” | Interest in knowing |
|  | Behavior changes from knowing risk |  | Any response that mentions a change in behavior (positive or negative) from knowing one’s risk for BC. | “If I knew my risk, I would be able to change my habits to prevent breast cancer.” | Behavior changes from knowing |
|  | Preferences in knowing risk |  | Mention of how they would prefer to know risk. | “I would prefer to do the BRCA in person.” | Preferences (risk) |
| Disseminating risk assessment |  |  | How information is delivered to the public | Statements on how to deliver the risk assessment. | Disseminating risk assessment |
|  | Strategies |  | Any mention of how to do risk assessments in community or clinical settings. | “I would share the BCRA with my friends when we see each other.” | Strategies (risk assessment) |
|  | Acceptability of online tool |  | Statements regarding the acceptability of the BCRA tool. | Statements regarding the acceptability of the BCRA tool. | Acceptability of online tool (risk assessment) |
|  |  | Individual | Responses to the question about using the tool. | “I would like this tool.” | Individual |
|  |  | Community | Responses to if women in community would find the form to be useful or if there would be interest. | “I do not think others would like this tool.” | Community |
|  | Barriers to risk assessment |  | Any mention of challenges or barriers to getting women to complete risk assessment outside clinical settings. | “I think some women can do the risk assessment if they do not have access to the internet.” | Barriers (risk assessment) |
|  | Preferences for risk assessment and communication |  | Any mention of preferences for risk communication and assessment delivery. | “I would rather do the risk assessment with my doctor than alone.” | Communication preferences (risk assessment) |
| Trusted sources of medical information |  |  | Any mention of where they receive medical information. | “I get my medical information from social media.” | Trusted sources |
| Social Support |  |  | Mention of social influences, community decision making, discussions about breast cancer. | “My sister always tells me to get screened.” | Social Support |
| Health literacy |  |  | Mentions of knowledge of cancer, terminology, etc. | “I have not heard of lifetime risk score.” | Health literacy |
| Other Health Comorbidities |  |  | Mentions of other comorbidities | “I take care of myself because my mom had lung cancer” | Other Health Comorbidities |
| Vignette responses |  |  |  |  | Vignette |
|  | Advice |  | Responses to what advice they would give if Ms. Hernandez was a friend | “I would tell Ms. Hernandez to get screened.” | Advice |
|  | Interest in knowing risk |  | Responses to if Ms. Hernandez would be interested in knowing risk | “I think Ms. Hernandez would be interested in knowing her risk.” | Vignette (interest) |
|  | Completing tool |  | Responses to if Mrs. Hernandez would complete the online tool | “I think Ms. Hernandez would not be interested in the tool.” | Vignette (use tool) |
|  | Facilitators to complete tool |  | Responses to what would make Mrs. Hernandez likely to complete the online tool | “Ms. Hernandez would likely use the tool.” | Vignette (tool facilitators) |
|  | High risk,  no symptoms |  | Responses to what Mrs. Hernandez should do next. | “Ms. Hernandez should talk to a doctor.” | High risk (no symptoms) |
|  | High risk,  symptoms |  | Responses to what Mrs. Hernandez should do next. | “Ms. Hernandez should get screened.” | High risk (symptoms) |
|  | Low risk,  no symptoms |  | Responses to what Mrs. Hernandez should do next. | “Ms. Hernandez does not have to do anything if she has no symptoms.” | Low risk (no symptoms) |
|  | Low risk,  symptoms |  | Responses to what Mrs. Hernandez should do next. | “Ms. Hernandez should see her doctor.” | Low risk (symptoms) |
| Care, values, and desires |  |  | Anything that mentions seeking information, their values, their care and desires. Not limited to breast cancer screening but general health | “I check my health for the sake of being healthy for my children.” | Care, values, and desires |
| Psycho-social factors |  |  | Anything that mentions emotions, fears, social or psychological influences not limited to the act of receiving a mammogram | “I am terrified of testing positive for breast cancer and dying.” | Psycho-social factors |
| Exemplar Quotes |  |  | Any strong quotes that summarize themes well. |  | Quotes |
| Unsure |  |  | We think the information is important but cannot find a code. Things we need to resolve. |  | Question |
